# Engineering the Host‐Graft Interface Stabilizes Vascular Integration and Improves Endocrine Function of Transplanted Ovarian Tissue

**DOI:** 10.1002/advs.78523

**Published:** 2026-11-11

**Authors:** Despina I. Pavlidis, Monica A. Rionda, Chloe E. Fischer, Maria A. Jennings, Delaney S. Sinko, Bhavya Vats, Emily E. Thomas, Margaret A. Brunette, Sasha Cai Lesher‐Pérez, Vasantha Padmanabhan, Brendon M. Baker, Ariella Shikanov

**Affiliations:** ^1^ Department of Biomedical Engineering University of Michigan Ann Arbor Michigan United States of America; ^2^ Division of Pediatric and Adolescent Gynecology Eunice Kennedy Shriver National Institute of Child Health and Human Development Bethesda Maryland United States of America; ^3^ Department of Chemical Engineering University of Michigan Ann Arbor Michigan United States of America; ^4^ Biointerfaces Institute University of Michigan Ann Arbor Michigan United States of America; ^5^ Department of Obstetrics and Gynecology University of Michigan Ann Arbor Michigan United States of America; ^6^ Department of Pediatrics University of Michigan Ann Arbor Michigan United States of America; ^7^ Department of Molecular & Integrative Physiology University of Michigan Ann Arbor Michigan United States of America; ^8^ Department of Macromolecular Science and Engineering University of Michigan Ann Arbor Michigan United States of America; ^9^ Cellular and Molecular Biology Program University of Michigan Ann Arbor Michigan United States of America

**Keywords:** biomaterial, hydrogel scaffold, medicine, ovary, peg hydrogel, revascularization, transplantation

## Abstract

Ovarian tissue cryopreservation and transplantation (OTCT) restores fertility and ovarian endocrine function in patients exposed to gonadotoxic cancer therapies but graft outcomes remain variable due to the avascular nature of the transplantation procedure. Here, the host‐graft interface is engineered using a synthetic microporous annealed particle (MAP) hydrogel transplantation strategy to support vascular integration of human ovarian tissue cubes. In a xenograft model of primary ovarian insufficiency (POI), non‐encapsulated ovarian tissue strips exhibit early but transient and disorganized host vascular infiltration. In contrast, tissue cubes encapsulated within MAP hydrogels (OvaMAPs) demonstrate sustained host vascular integration with significantly greater mouse vessel area and length at 3 and 6 weeks post‐transplantation. By 20 weeks, both non‐encapsulated grafts and OvaMAPs restore circulating estradiol and suppress follicle‐stimulating hormone, confirming integration of the grafts with the host hypothalamic‐pituitary axis. Notably, OvaMAP recipients exhibit reduced variability in estradiol levels, suggesting consistent, predictable graft function. These findings identify stabilization of vascular remodeling at the host‐graft interface as a key determinant of ovarian graft performance and demonstrate that a fully synthetic microporous biomaterial can improve the durability and predictability of endocrine function restoration after OTCT for patients at risk of POI.

## Graphical abstract

Conventional transplantation of human ovarian tissue results in early vascular infiltration that is not sustained over time. Engineering the host‐graft interface by combining multiple, smaller ovarian tissue pieces with a microporous annealed particle (MAP) hydrogel instead supports sustained tissue revascularization and perfusion. This OvaMAP transplantation strategy also supports more consistent long‐term endocrine function, highlighting its potential for improving transplantation outcomes.

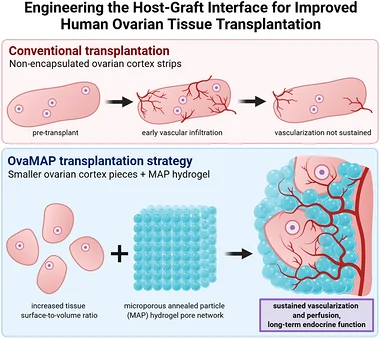

## 1 Introduction

Primary ovarian insufficiency (POI), a condition defined as the loss of ovarian activity before the age of 40, affects approximately 68% of patients with ovaries treated with gonadotoxic cancer therapies [1, 2]. The consequences of POI include not only premature infertility but also ovarian endocrine dysfunction, which is associated with increased risk of cardiovascular disease, osteoporosis, mood disorders, and cognitive decline [1, 3]. Clinical management of POI in cancer survivors therefore focuses on preserving fertility and restoring gonadal hormone levels. The current standard of care for the latter is hormone replacement therapy (HRT). This therapy, however, supplies only estradiol and progesterone at levels that are not personalized to the patient [4]. Ovarian tissue cryopreservation and transplantation (OTCT) – the cryopreservation of a patient's ovarian tissue prior to cancer treatment followed by transplantation after remission – is the only clinical intervention capable of restoring both fertility and physiological ovarian endocrine function [5]. OTCT accomplishes this by retaining the dynamic ovarian hormone levels which arise from physiological crosstalk between the hypothalamic‐pituitary‐ovarian axis and other organs and tissues of the endocrine system. Since the first report of OTCT in 2000 [6], this procedure has shown promising outcomes with at least 189 live births documented [7] and ovarian endocrine function restored in approximately 89% of patients 4 to 5 months after tissue transplantation [8]. Despite these encouraging results, the live birth rate remains low at just 28%, and the duration of graft function is prohibitively short, ranging from 0.7 to 5 years [7].

The limited and variable success of OTCT is attributed to both the inherent heterogeneity of human ovarian tissue [9] and the avascular nature of the transplantation procedure [10]. Follicle number and distribution vary widely between patients and even within a single ovary. This inter‐ and intra‐patient heterogeneity, coupled with the lack of non‐destructive methods for quantifying follicle content at the time of tissue cryopreservation and transplantation, results in a limited ability to predict ovarian graft lifespan and the likelihood of pregnancy. Furthermore, ovarian tissue lacks sufficiently large blood vessels for surgical vascular anastomosis, which makes restoration of blood flow entirely dependent on post‐transplantation angiogenesis [10]. Human ovarian xenograft studies have primarily characterized the early post‐transplantation period, demonstrating that grafts remain hypoxic and that host‐derived revascularization occurs over a 10‐day window [11, 12], leading to ischemic tissue injury and follicle loss. The combination of these factors leads to shortened, unpredictable graft function, which poses a considerable clinical challenge given that each patient only has a finite amount of their own cryopreserved tissue available.

Accordingly, a major focus for improving ovarian graft function has been the development of strategies to support graft vascular integration following avascular tissue transplantation. Within the context of human ovarian xenograft studies, naturally derived biomaterials, including fibrin [13, 14, 15, 16, 17, 18, 19], Matrigel [20, 21], and gelatin [22], have shown promise as supportive scaffolds for transplantation. More recently, however, granular hydrogels composed of micron‐scale hydrogels (“microgels”) have emerged as versatile scaffolds for improving endogenous tissue repair across a breadth of tissues [23]. Microporous annealed particle (MAP) hydrogels [24], a subclass of granular hydrogels, feature secondary crosslinks between their microgel subunits which produce a uniform, interconnected cell‐scale porous network that facilitates infiltration of host cells and molecular diffusion essential to the function and survival of constituent cells [25]. The modularity of the microgel subunits enables precise control over scaffold porosity, composition, and functionality, which has proven effective in promoting vascular infiltration in vivo [26, 27, 28, 29, 30, 31, 32, 33], making MAP hydrogels promising candidates for supporting vascular integration of transplanted ovarian tissue.

Here, we utilized a MAP hydrogel scaffold to encapsulate and transplant human ovarian tissue. Guided by clinical recommendations that emphasize high tissue surface‐to‐volume ratio for graft survival [34], we hypothesized that encapsulating multiple small pieces of ovarian tissue in a MAP hydrogel would support graft vascular integration and long‐term function. To investigate this hypothesis, we transplanted human ovarian tissue pieces encapsulated in MAP hydrogels (OvaMAPs) into ovariectomized mice and analyzed graft revascularization, reperfusion, and restoration of ovarian endocrine function at early (3 days and 1 week) and late (3, 6, and 20 weeks) post‐transplantation time points. Our findings demonstrate that the OvaMAP transplantation strategy, which combines MAP hydrogel encapsulation with smaller ovarian tissue pieces, improved graft vascular integration, perfused blood vessel formation, and hormone function compared to conventional non‐encapsulated tissue strip transplantation or bulk hydrogel encapsulation of small tissue pieces. To our knowledge, this study is the first to utilize MAP hydrogels as a whole tissue transplantation scaffold, leveraging the interconnected microporous architecture of MAP hydrogels to support functional revascularization of transplanted human ovarian tissue.

## 2 Results

### 2.1 Non‐Encapsulated Ovarian Tissue Exhibits Transient Revascularization

To establish a baseline control reflecting the current clinical avascular transplantation practice, we first investigated revascularization of non‐encapsulated human ovarian grafts transplanted into immunodeficient ovariectomized mouse hosts. Ovarian cortex from donated human ovaries was processed into 10 × 10 × 1 mm (length × width × height) squares and then cryopreserved using a slow freezing protocol (Figure 1A). To prepare the tissue for transplantation, ovarian cortex squares were thawed, cut into approximately 20 strips (5 × 1 × 1 mm each), and then implanted into the parametrial fat pads of ovariectomized NOD.Cg‐*rkdc^scid^ Il2rg^tm1Wjl^*/SzJ (NSG) female mice.

**FIGURE 1:**
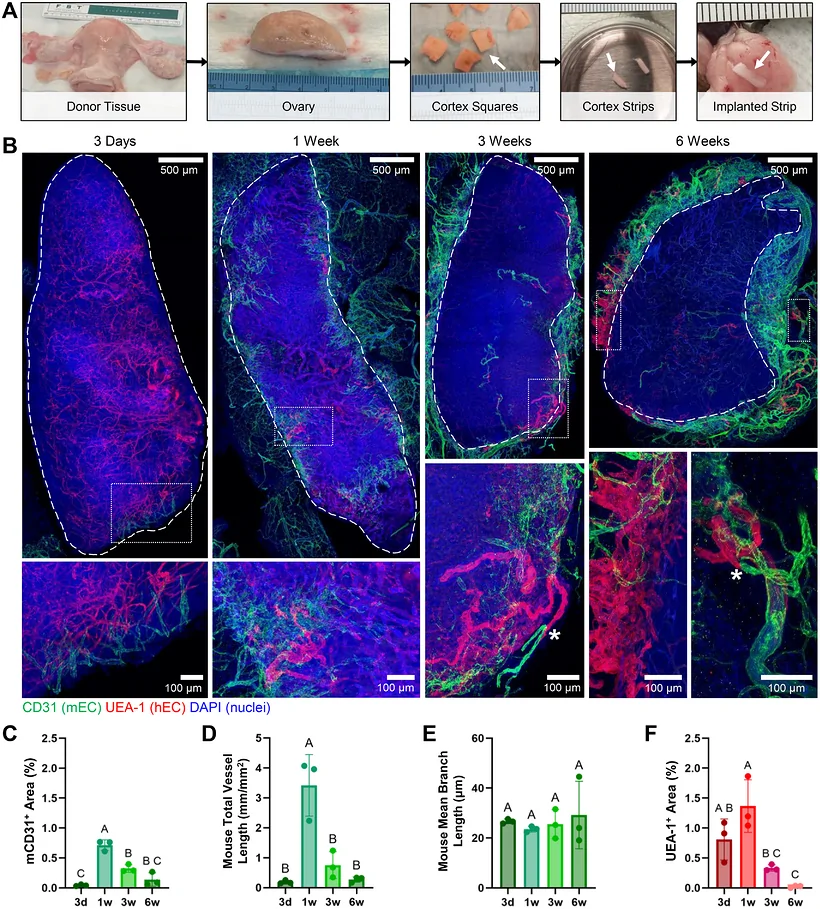
Revascularization of Non‐Encapsulated Human Ovarian Tissue Grafts. (A) Human ovarian donor tissue processing into cortex squares prior to cryopreservation. Upon thawing, ovarian cortex squares were cut into strips and implanted into the parametrial fat pads of immune‐deficient, ovariectomized mice. Arrows mark the human ovarian tissue. (B) Representative maximum intensity projection images show post‐implantation mouse (CD31, green) and human (UEA‐1, red) vasculature dynamics in human ovarian tissue strips over the course of 6 weeks. Dashed white lines delineate the border of the implanted human tissue within the surrounding mouse fat pad. Asterisks indicate chimeric (UEA‐1^+^/CD31^+^) vasculature. (C) Percent area of implanted human ovarian tissue strip that stained positively for mouse vasculature. (D) Mouse total vessel length per tissue strip area. (E) Mouse mean branch length within tissue strip. (F) Percent area of implanted human ovarian tissue strip that stained positively for human vasculature. Data points represent individual grafts from distinct mouse hosts (n = 3 per time point). Data analyzed by one‐way ANOVA with Tukey's multiple comparisons test, *p* < 0.05, different letters indicate statistical significance.

Transplanted tissue strips were explanted at 3 days and 1, 3, and 6 weeks to investigate the temporal dynamics of non‐encapsulated graft revascularization. The explants were first optically cleared to facilitate downstream imaging and then stained with species‐specific vascular markers. Mouse vasculature was labeled with a mouse‐specific CD31 antibody, and human vasculature was labeled with Ulex europaeus agglutinin 1 (UEA‐1) (Figure 1B). Confocal imaging revealed mouse vasculature infiltration in the human ovarian grafts as early as 3 days post‐transplantation, which increased 17‐fold by 1 week (Figure 1C). By 3 weeks, however, mouse CD31^+^ area within the grafts decreased significantly, dropping to less than half of the percent area observed at 1 week. Subsequently, mouse vasculature within the grafts further declined by 6 weeks, returning to the baseline percent area observed at the initial 3‐day time point. Total mouse vessel length per ovarian graft area demonstrated a similar temporal pattern, with a transient peak at 1 week followed by decreases at 3 and 6 weeks (Figure 1D). In contrast, the mean branch length of mouse vessels within the ovarian grafts remained similar at all time points (Figure 1E), suggesting that the observed changes in mouse vasculature area within the grafts over time were likely driven by variations in the number of infiltrating vessels rather than by the branch length of existing vessels.

Native human vasculature within the grafts remained stable during the first week but decreased significantly at 3 and 6 weeks (Figure 1F). Positive UEA‐1 staining and UEA‐1^+^/CD31^+^ chimeric vessels in the mouse tissues surrounding the grafts after 3 and 6 weeks post‐transplantation indicated outgrowth and infiltration of human endothelial cells (ECs) into the host mouse fat pads (Figure 1B). Overall, these results suggested that the host‐graft interface established through non‐encapsulated tissue strip transplantation enabled only transient mouse vascular infiltration, failing to support stable, long‐term host‐derived graft revascularization.

### 2.2 Development of a MAP Hydrogel for Ovarian Tissue Encapsulation and Transplantation

The transient revascularization observed in the non‐encapsulated grafts prompted the development of a transplantation strategy to engineer the host‐graft interface. Specifically, we combined smaller ovarian tissue pieces – increasing the tissue surface area available for interaction with the scaffold and host environment – with a MAP hydrogel designed to provide an interconnected microporous network for host infiltration. Poly(ethylene glycol) (PEG) MAP hydrogels were formed with the plasmin‐cleavable peptide YKNS and functionalized with the integrin‐binding peptide RGD (Figure 2A). YKNS was incorporated to enable local ovarian tissue‐driven remodeling via plasmin activity during follicle development [35], with prior work establishing its use for follicular remodeling [36, 37] and human ovarian tissue xenografts [38]. This formulation was used to generate microgels in a step emulsification microfluidic device. Once swollen and equilibrated, microgels measured 90 ± 5 µm (mean ± SD) in diameter.

**FIGURE 2:**
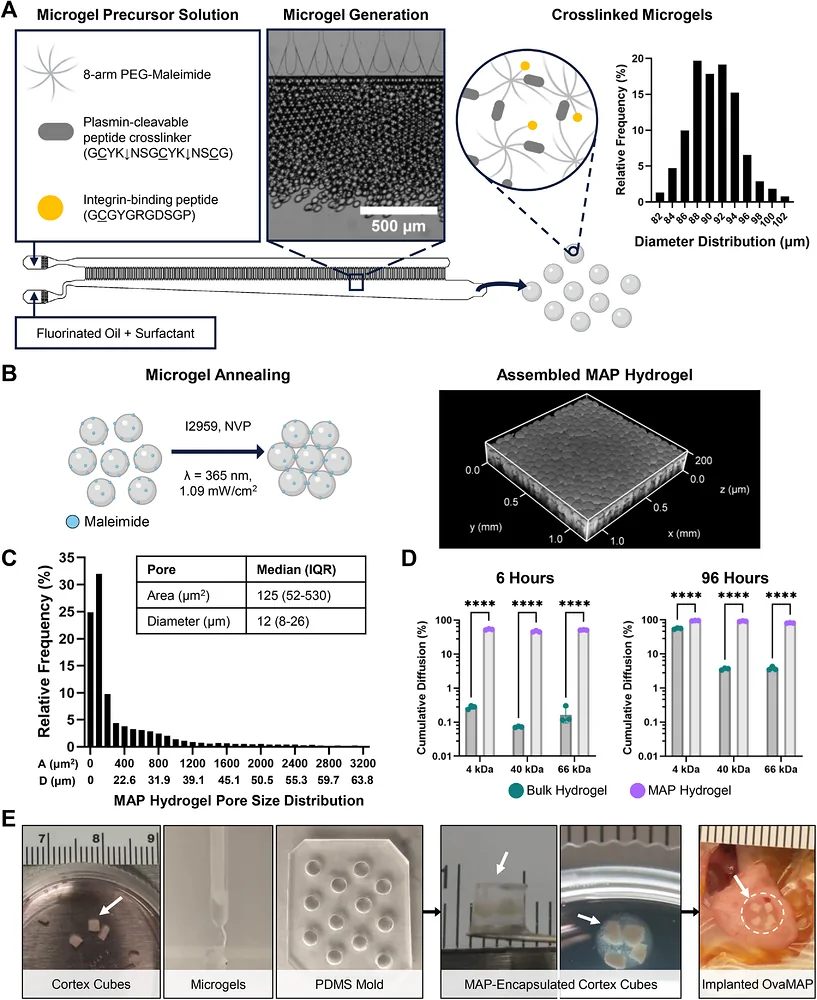
MAP Hydrogel Fabrication and Characterization. (A) Microfluidic fabrication of PEG microgels and diameter distribution of crosslinked microgels. (B) Microgels were annealed through photopolymerization of remaining maleimide groups, producing an assembled MAP hydrogel shown in the representative 3D rendering. (C) MAP hydrogel pore area distribution was measured using confocal images of assembled MAP hydrogels (n = 5 MAP hydrogels). Pore diameter distribution was estimated by treating pore areas as corresponding to areas of circles. (D) In vitro cumulative diffusion of 4 kDa and 40 kDa FITC‐DEX and 66 kDa Alexa Fluor 488‐conjugated BSA through bulk (n = 3) and MAP (n = 3) hydrogels after 6 h and 96 h, demonstrating greater molecular transport through MAP hydrogels. Each data point represents one hydrogel. Data analyzed by two‐way ANOVA with Šidák's multiple comparisons test, *p* < 0.05, significant differences indicated as ^****^ *p* ≤ 0.0001. (E) Encapsulation of human ovarian cortex cubes within MAP hydrogels and implantation of OvaMAP into the mouse parametrial fat pad. Arrows mark the ovarian tissue and OvaMAP.

When formulating the microgels, a subset of maleimide (MAL) functional groups were intentionally left unreacted. These residual MAL groups enabled subsequent annealing of microgels into the MAP hydrogel via ultraviolet light photoinitiated radical polymerization (Figure 2B) and rendered individual microgels as well as the assembled MAP hydrogel semi‐degradable. To assess MAP hydrogel porosity, microgels were labeled with a fluorophore and imaged by confocal microscopy (Figure 2B). Analysis of confocal images (Figure S1A) revealed a median pore area of 125 µm^2^ (IQR: 52–530 µm^2^) (Figure 2C). By treating these pore areas as circles, the assembled MAP hydrogel was found to have a median pore diameter of 12 µm (IQR: 8–26 µm), confirming the formation of micron‐scale void spaces suitable for cellular infiltration. In contrast, bulk hydrogels formed using the same formulation had a mesh size of 131 ± 0.4 nm (mean ± SD), confirming nanoscale porosity typical of bulk hydrogels (Figure S1B). Hydrogel mechanical properties were characterized using shear rheology (Figure S2). Bulk hydrogels exhibited a storage modulus of 5062 ± 226 Pa compared to 2705 ± 259 Pa for MAP hydrogels. Bulk hydrogels prepared using the microgel formulation exhibited a mean storage modulus of 721 ± 141 Pa, providing an approximation of the mechanical properties of the constituent microgels. Significant differences in storage modulus were observed between all three hydrogel formulations.

Next, mass transport through the MAP hydrogel's porous network was examined by utilizing fluorescently tagged molecules and in vitro diffusion assays (Figure S1C). Specifically, we selected Alexa Fluor 488‐conjugated bovine serum albumin (BSA) as a representative globular protein as well as FITC‐conjugated dextran (DEX) of various molecular weights. Transwell inserts were used to assess the diffusion of these molecules across MAP and bulk hydrogels. Over a period of 96 h, all tested molecules (4 kDa DEX, 40 kDa DEX, 66 kDa BSA) exhibited significantly greater diffusion through the MAP hydrogel compared to the bulk hydrogel (Figure S1D–F). After 6 h, close to half of the solutes had already diffused through the MAP hydrogel. The cumulative diffusion through MAP hydrogels was 193 times greater for 4 kDa DEX, 633 times greater for 40 kDa DEX, and 282 times greater for BSA compared to bulk hydrogels after 6 h (Figure 2D). Moreover, after 96 h, most of the solutes had diffused through the MAP hydrogels. Accordingly, the cumulative diffusion through MAP hydrogels was 1.7 times greater for 4 kDa DEX, 25 times greater for 40 kDa DEX, and 21 times greater for BSA compared to bulk hydrogels after 96 h.

### 2.3 OvaMAPs Stabilize Human Ovarian Tissue Revascularization

Next, we evaluated the capacity of the OvaMAP transplantation strategy to support stable graft revascularization. Cryopreserved human ovarian tissue squares were thawed and manually cut into cubes measuring ∼1 mm in width and length to increase tissue surface‐to‐volume ratio by approximately 30%. Microgels were pipetted as two layers into a polydimethylsiloxane (PDMS) mold, with human ovarian tissue cubes embedded in between the microgel layers. This construct was then exposed to ultraviolet light to anneal the microgels, resulting in human ovarian tissue cubes fully encapsulated within a MAP hydrogel (OvaMAP). Following the same approach used for the non‐encapsulated ovarian tissue strips, we then transplanted OvaMAPs into ovariectomized NSG female mice (Figure 2E).

Confocal imaging of OvaMAPs (Figure 3A) revealed that the onset of mouse vasculature infiltration occurred between 1 and 3 weeks post‐transplantation. Quantitative analyses of mouse vasculature within ovarian tissue cubes demonstrated that mouse CD31^+^ area (Figure 3B), total vessel length (Figure 3C), and mean branch length (Figure 3D) increased significantly by 3 weeks and remained stable through 6 weeks, suggesting stable host revascularization of the human ovarian tissue cubes within OvaMAPs.

**FIGURE 3:**
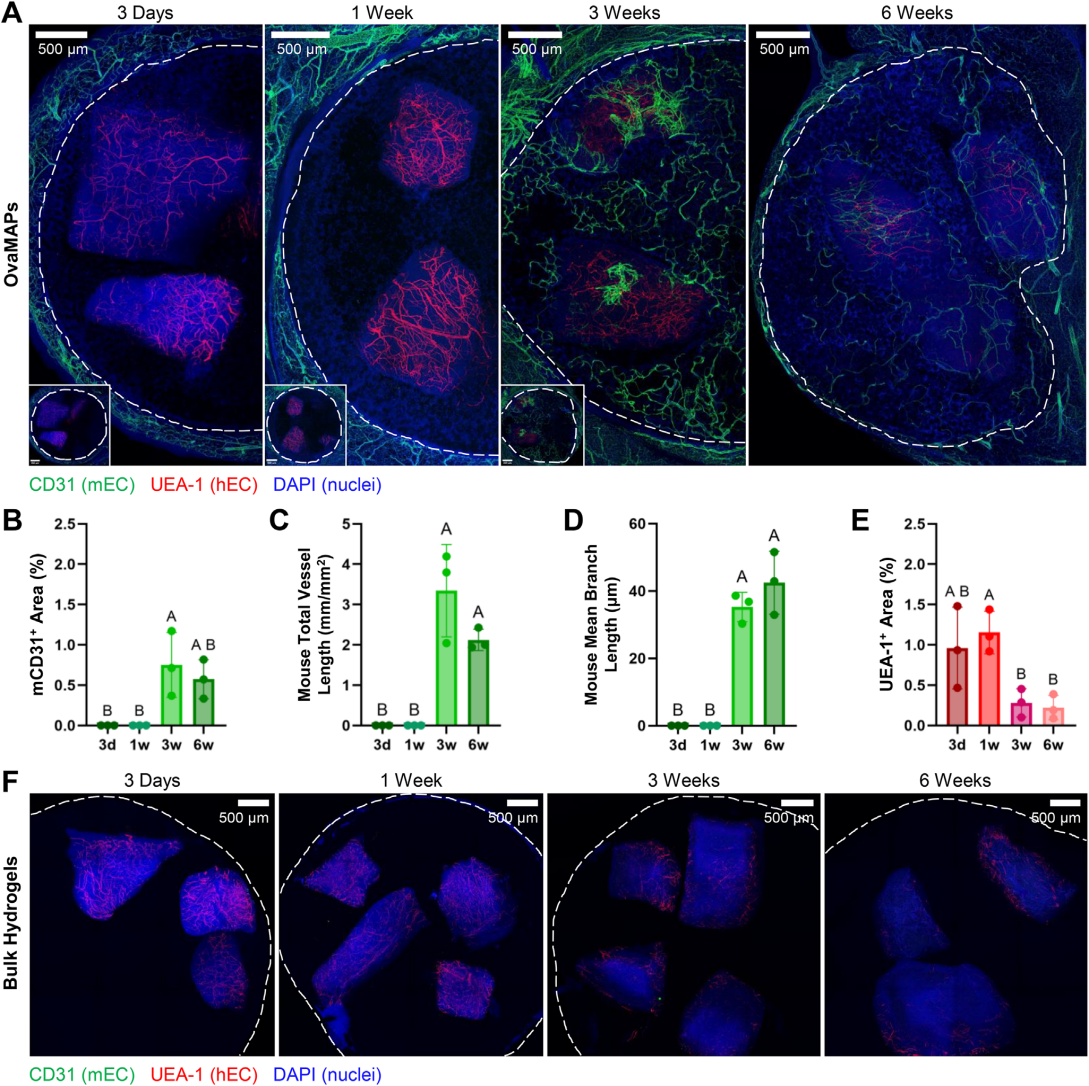
Revascularization of MAP Hydrogel‐Encapsulated Human Ovarian Tissue Grafts. (A) Representative maximum intensity projection images show post‐implantation mouse (CD31, green) and human (UEA‐1, red) vasculature dynamics in human ovarian tissue cubes encapsulated in MAP hydrogels (OvaMAPs) over the course of 6 weeks. Dashed white lines delineate the OvaMAP border within the surrounding mouse fat pad. Insets in the bottom left display the entire OvaMAP graft. (B) Percent area of OvaMAP human ovarian tissue cubes that stained positively for mouse vasculature. (C) Mouse total vessel length per OvaMAP total tissue cube area. (D) Mouse mean branch length within OvaMAP tissue cubes. (E) Percent area of OvaMAP human ovarian tissue cubes that stained positively for human vasculature. (F) Representative maximum intensity projection images show post‐implantation mouse (CD31, green) and human (UEA‐1, red) vasculature dynamics in human ovarian tissue cubes encapsulated in bulk hydrogels over the course of 6 weeks. Dashed white lines delineate the bulk hydrogel border. Note the lack of mouse CD31^+^ staining and the lack of mouse fat pad integration, suggesting the bulk hydrogel did not support mouse vascular infiltration at any time point. Data points represent individual grafts from distinct mouse hosts (n = 3 per time point). Data analyzed by one‐way ANOVA with Tukey's multiple comparisons test, *p* < 0.05, different letters indicate statistical significance.

Similar to the non‐encapsulated grafts, native human vasculature in the ovarian tissue cubes within OvaMAPs regressed over time. Initially, native human vasculature remained stable between 3 days and 1 week but decreased by 3 and 6 weeks (Figure 3E). Nonetheless, OvaMAPs retained approximately tenfold greater native human vasculature compared to the non‐encapsulated grafts.

As a non‐microporous control for OvaMAPs, ovarian tissue cubes were encapsulated in bulk nanoporous PEG hydrogels and transplanted under identical conditions. Confocal imaging of these bulk hydrogel grafts showed similar regression of native human vasculature over time (Figure S4). Importantly, bulk hydrogels showed no evidence of mouse vasculature infiltration up to 6 weeks post‐transplantation (Figure 3F).

### 2.4 Engineering the Host‐Graft Interface Sustains Long‐Term Tissue Revascularization and Reperfusion

We next compared graft revascularization between OvaMAPs and conventionally transplanted non‐encapsulated tissue strips, representing distinct transplantation strategies and associated host‐graft interfaces. DAPI^+^ cells were present within the MAP hydrogel pores at 3 days and 1 week (Figure 4A). Non‐encapsulated grafts showed early mouse vasculature infiltration that increased by 1 week but remained disorganized and primarily localized to the graft periphery (Figure 4B). Accordingly, quantitative comparisons of mouse CD31^+^ area (Figure 4C) and mouse total vessel length (Figure 4D) demonstrated no differences between OvaMAPs and non‐encapsulated grafts at 3 days. At 1 week, non‐encapsulated grafts exhibited greater mouse CD31^+^ area and total vessel length compared to OvaMAPs.

**FIGURE 4:**
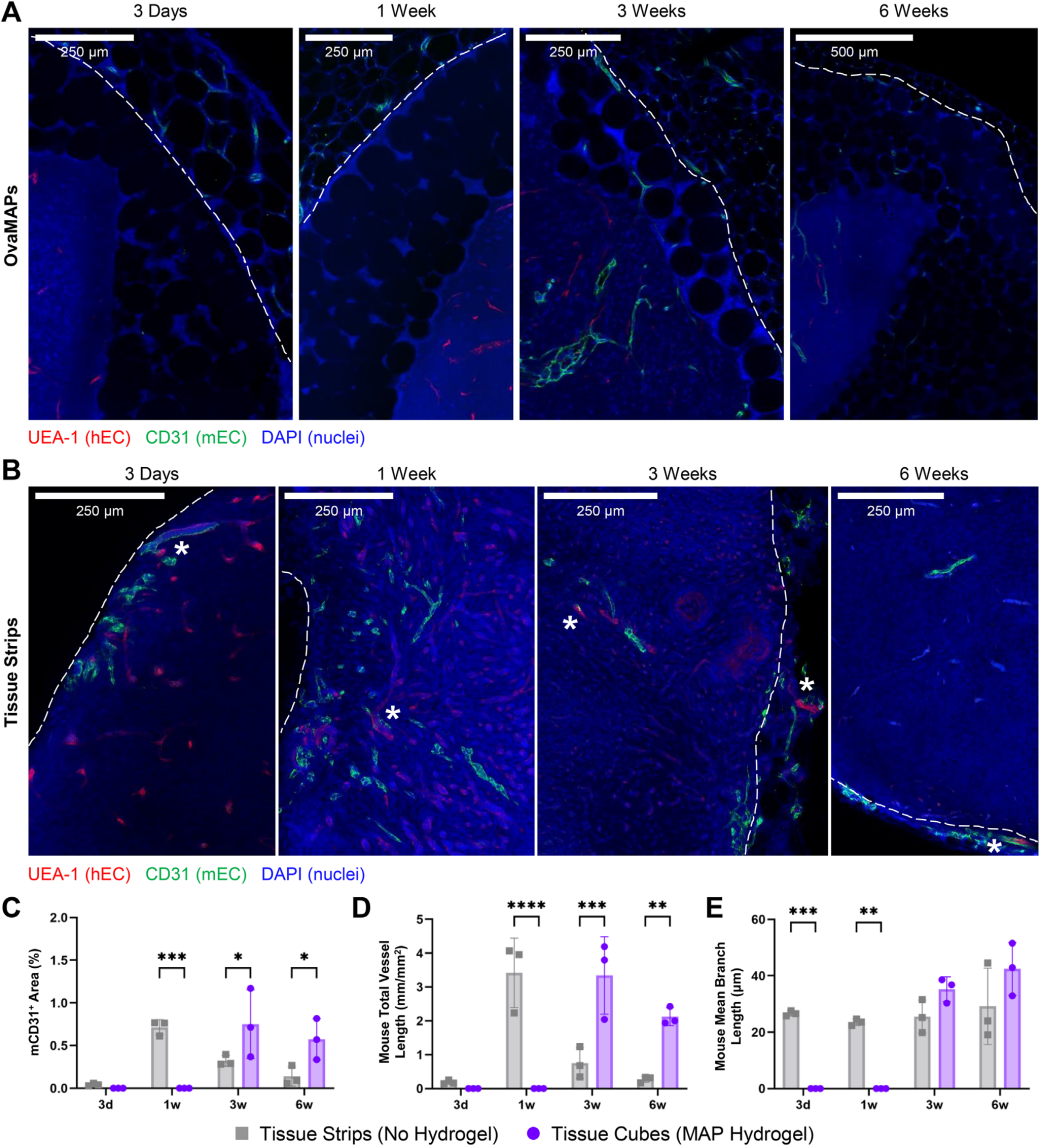
OvaMAP Sustains Long‐Term Human Ovarian Graft Revascularization. (A) Representative z‐slice images demonstrate progressive cellular infiltration into OvaMAPs from 3 days to 6 weeks post‐transplantation. Early DAPI^+^ cell migration into MAP hydrogel pores was observed by 3 days, followed by infiltration of mouse CD31^+^ endothelial cells by 3–6 weeks, which localized to the encapsulated ovarian tissue. Dashed white lines delineate the OvaMAP border within the surrounding mouse fat pad. (B) Representative z‐slice images of non‐encapsulated tissue strips depicting mouse vasculature infiltration into tissue grafts over the course of 6 weeks. Dashed white lines delineate the border of the implanted human tissue within the surrounding mouse fat pad. Asterisks indicate chimeric (UEA‐1^+^/CD31^+^) vasculature. (C) Comparison of the percent area of implanted human ovarian tissue that stained positively for mouse vasculature. (D) Comparison of mouse total vessel length per tissue strip area or per total tissue cube area (OvaMAPs). (E) Comparison of mouse mean branch length within tissue strip or tissue cubes (OvaMAPs). Data points represent individual grafts from distinct mouse hosts (n = 3 per time point, per condition). Data analyzed by two‐way ANOVA with Šidák's multiple comparisons test, *p* < 0.05, significant differences indicated as ^*^ *p* ≤ 0.05, ^**^ *p* ≤ 0.01, ^***^ *p* ≤ 0.001.

By 3 weeks, magnified images of OvaMAPs revealed mouse vasculature within the surrounding fat pad, throughout the MAP hydrogel microporous network, and within the encapsulated human ovarian tissue cubes (Figure 4A). In contrast, non‐encapsulated tissue strips revealed a dramatic decrease in mouse vasculature infiltration (Figure 4B). Quantitative comparisons demonstrated approximately twofold greater mouse CD31^+^ area in OvaMAPs than in non‐encapsulated grafts (Figure 4C). Additionally, mouse total vessel length in OvaMAPs was approximately 4 times greater than in non‐encapsulated grafts (Figure 4D). By 6 weeks, these differences were even more pronounced as OvaMAPs displayed an approximately fourfold greater mouse CD31^+^ area and sevenfold greater mouse total vessel length relative to non‐encapsulated grafts. Moreover, analysis of mouse mean branch length (Figure 4E) revealed that, although non‐encapsulated grafts showed significantly longer mean branch lengths than OvaMAPs at 3 days and 1 week, this early advantage was not sustained and lost statistical significance by 3 and 6 weeks.

To assess the functionality of infiltrating mouse vasculature, we stained 3‐ and 6‐week time point OvaMAPs with a mouse red blood cell (RBC) marker (TER‐119). At 3 and 6 weeks, numerous mouse vessels containing mouse RBCs were present throughout the MAP hydrogels’ pores and within the encapsulated human ovarian tissue cubes (Figure 5A). Higher magnification images revealed functional mouse vasculature infiltrating between the OvaMAP microgel subunits (Figure 5Bi, iii), suggesting that the interstitial pore space guided the ingrowth of vascular structures. Functional mouse vessels were also identified within the encapsulated tissue cubes (Figure 5Bii, iv), confirming that the human ovarian tissue received blood flow throughout the later time points.

**FIGURE 5:**
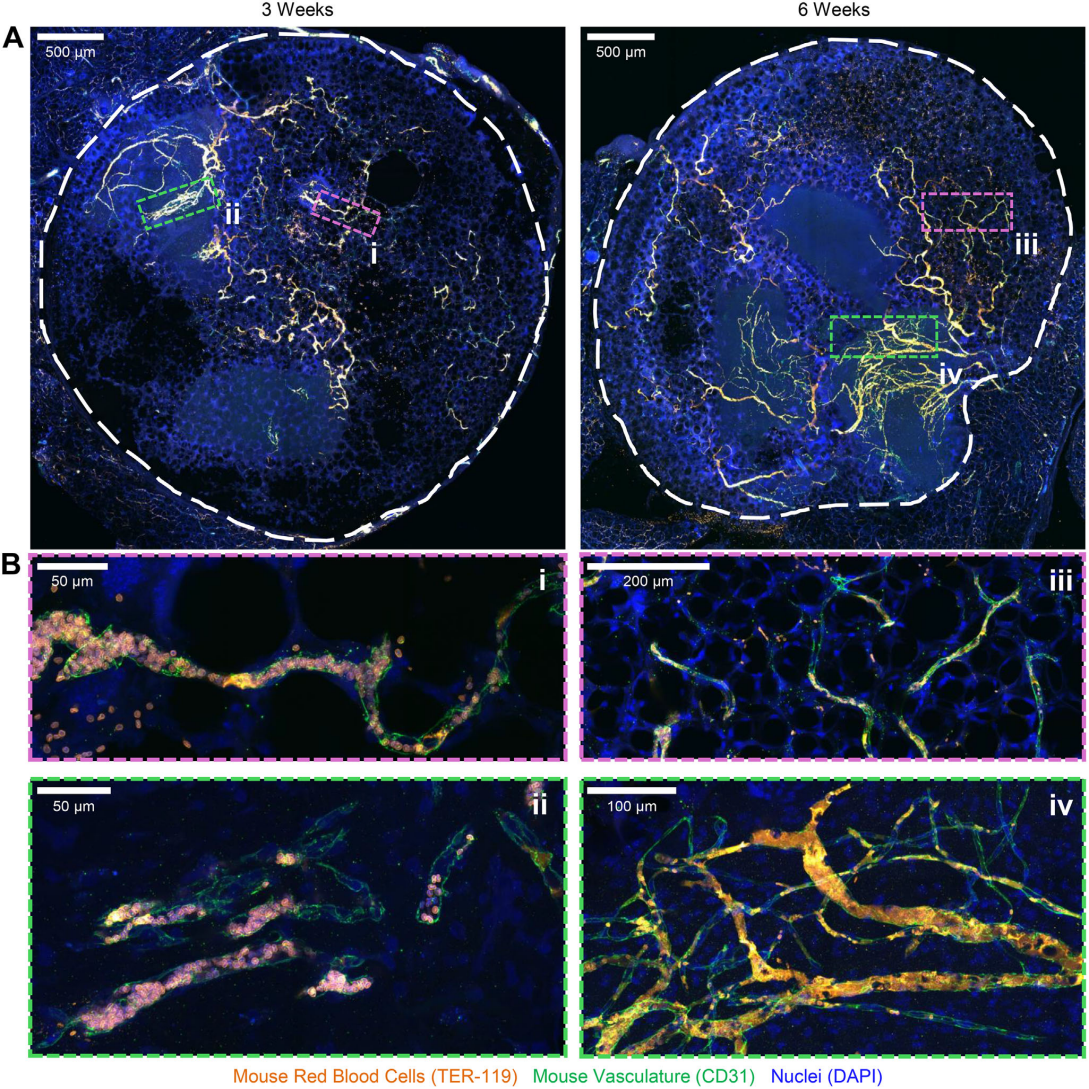
Perfused, Functional Host Revascularization of OvaMAPs. (A) Infiltrating mouse host vasculature (CD31, green) in OvaMAPs 3 and 6 weeks post‐transplantation contains mouse red blood cells (TER‐119, orange), confirming functional perfusion. (B) Perfused vasculature was evident both within MAP hydrogel pores (i, iii) and within the encapsulated tissue cubes (ii, iv) at 3 and 6 weeks post‐transplantation. Dashed white lines delineate the OvaMAP border within the surrounding mouse fat pad. Magenta dashed lines delineate regions containing perfused vasculature within hydrogel pores, and green dashed lines delineate regions containing perfused vasculature within encapsulated ovarian tissue cubes.

### 2.5 Hydrogel‐Encapsulated Ovarian Tissue Restores Dynamic Ovarian Endocrine Function

Cryopreserved ovarian cortical tissue primarily contains immature follicles that require approximately 4 to 5 months to reach hormone‐producing stages [8]. Accordingly, we investigated graft hormone production and integration with both the host hypothalamic‐pituitary (HP) axis and host vasculature 20 weeks post‐transplantation using ovariectomized NSG female mice engrafted with human ovarian tissue delivered either as non‐encapsulated tissue strips or as cubes encapsulated in MAP or bulk hydrogels. Consistent with our previous analyses at 3 and 6 weeks, the 20‐week non‐encapsulated graft demonstrated minimal mouse vasculature infiltration, while OvaMAP exhibited sustained mouse vasculature infiltration localized to the encapsulated human ovarian tissue cubes (Figure 6A). Bulk hydrogels did not support mouse vasculature infiltration at any time point (Figures 3F and 6A).

**FIGURE 6:**
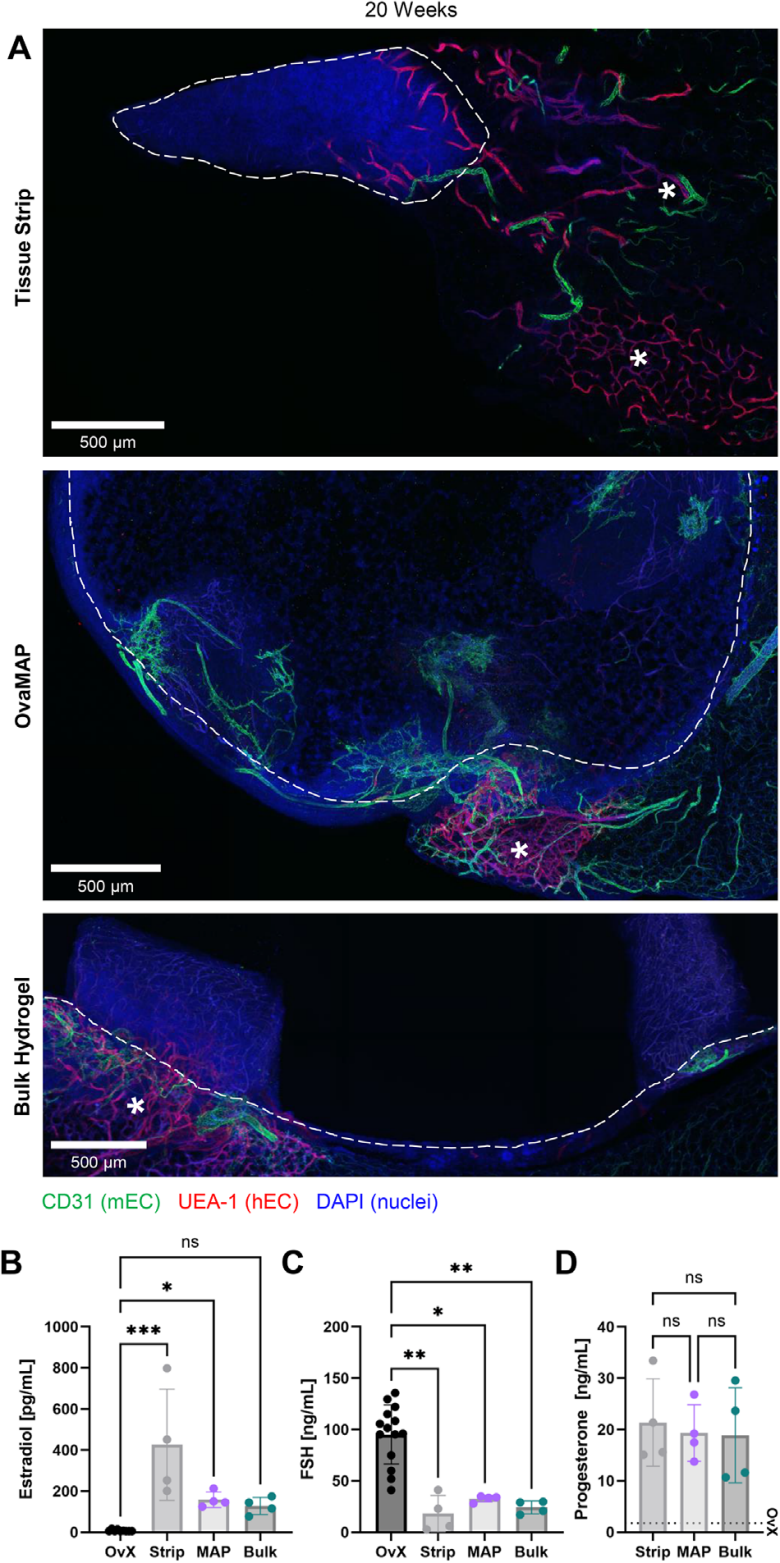
Long‐term Graft Revascularization and Endocrine Function Restoration. (A) Representative maximum intensity projection images show post‐implantation mouse (CD31, green) and human (UEA‐1, red) vasculature dynamics within grafts 20 weeks post‐transplantation. The non‐encapsulated tissue strip image comprises 6 z‐slices. The OvaMAP and bulk hydrogel graft comprise 101 z‐slices. Dashed white lines delineate graft borders within the surrounding mouse fat pad. Asterisks denote human UEA‐1^+^ vasculature in the adjacent host mouse tissue. At 20 weeks, serum estradiol (B), follicle‐stimulating hormone (FSH) (C), and progesterone (D) levels were quantified in host mice (n = 4 per condition) implanted with non‐encapsulated tissue strips, OvaMAPs, or bulk hydrogel‐encapsulated tissue cubes. The dashed line marks mean progesterone levels in ovariectomized NSG mice from a previously published study [39]. Each data point represents one host mouse. Data analyzed by Kruskal‐Wallis test with Dunn's multiple comparisons test, *p* < 0.05, significant differences indicated as ^*^ *p* ≤ 0.05, ^**^ *p* ≤ 0.01, ^***^ *p* ≤ 0.001.

Measurements of circulating hormone levels in the host mice revealed that both non‐encapsulated grafts and OvaMAPs produced significantly higher estradiol levels, 425.9 ± 270.5 pg/mL (mean ± SD) and 158.2 ± 37.91 pg/mL, respectively (Figure 6B), compared to ovariectomized control female NSG mice (7.608 ± 4.745 pg/mL). Furthermore, all transplant conditions exhibited significantly lower follicle‐stimulating hormone (FSH) levels compared to ovariectomized control female NSG mice (Figure 6C). In ovariectomized control mice, FSH levels reached 95.16 ± 28.79 ng/mL due to the lack of sufficient concentrations of circulating estradiol. In contrast, FSH for non‐encapsulated grafts, OvaMAPs, and bulk hydrogel grafts dropped to 18.21 ± 17.71 ng/mL, 32.61 ± 3.23 ng/mL, and 24.19 ± 6.27 ng/mL, respectively, suggesting integration of the grafts with the host HP axis and subsequent successful restoration of physiological negative feedback between estradiol and FSH.

Progesterone was also present and was not statistically different between the transplant conditions (non‐encapsulated grafts: 21.36 ± 8.52 ng/mL; OvaMAPs: 19.31 ± 5.49 ng/mL; bulk hydrogel grafts: 18.87 ± 9.24 ng/mL), but was multifold higher than reported progesterone levels for ovariectomized and even intact NSG female mice [39], suggesting the presence of luteal cells in the grafts capable of responding to circulating gonadotropins (Figure 6D). The detection of measurable progesterone levels further indicated that all three transplant conditions restored ovarian endocrine function by 20 weeks post‐transplantation.

## 3 Discussion

Restoration of durable ovarian graft function after OTCT remains limited by human ovarian tissue heterogeneity and ischemic tissue injury. In this study, engineering the host‐graft interface through the OvaMAP transplantation strategy fundamentally altered the trajectory of vascular integration of transplanted human ovarian tissue. Instead of the rapid but transient and disorganized vascular response observed with conventional transplantation of ovarian tissue strips, MAP hydrogel delivery of smaller tissue cubes produced organized, perfused, and sustained revascularization that supported long‐term endocrine function. These findings identify stabilization of vascular integration, rather than acceleration of early angiogenesis alone, as a critical determinant of graft performance, and introduce a fully synthetic, tunable platform for improving OTCT outcomes.

Current clinical practice involves the avascular transplantation of non‐encapsulated ovarian tissue, with the assumption that early host vessel infiltration will revascularize the graft and enable its long‐term function. Our temporal analysis, however, challenges this assumption. Non‐encapsulated tissue strips exhibited a surge in host vascular infiltration at 1 week, consistent with previous studies [20, 21, 40], but this response subsequently diminished by 3 to 6 weeks and was accompanied by sparse and disorganized vessels largely confined to the graft periphery. Prior xenograft studies have focused primarily on early (days [14, 15, 19, 21]) or late (months [17, 18]) endpoints; by resolving intermediate time points, our data reveal a previously underappreciated phase of vascular instability in OTCT. The observed angiogenic response likely reflects the formation of immature or poorly integrated vessels at the non‐encapsulated tissue strip interface that fail to remodel into a durable vascular network, providing a potential mechanistic explanation for the highly variable graft function duration noted clinically.

In contrast, MAP‐encapsulated tissue cubes exhibited progressive host vascular ingrowth that increased between 1 and 3 weeks and remained stable through later time points. Host vessels infiltrating OvaMAPs were perfused and extended through the interconnected pore network into the tissue cube core, resulting in substantially greater vessel area and length compared to non‐encapsulated grafts. As such, the OvaMAP platform supported the transition from vascular ingrowth to sustained vascular integration rather than a short‐lived burst of angiogenesis. Direct comparison of MAP and bulk hydrogels containing equivalently prepared tissue cubes further demonstrated the importance of scaffold properties within the engineered interface. Bulk nanoporous hydrogels failed to support vascular infiltration, supporting a role for MAP microporosity in stable, functional graft revascularization. MAP and bulk hydrogels also exhibited different mechanical properties, suggesting that scaffold mechanics may furthermore contribute to the vascular responses observed in these materials. Together, these findings demonstrate that the physical properties of the host‐graft interface can dictate the quality and persistence of vascular integration.

Several features of the MAP system likely contributed to the revascularization dynamics observed in the encapsulated ovarian tissue cubes. The interconnected cell‐scale pore network supported molecular transport and host cell infiltration, while plasmin‐sensitive microgel crosslinks enabled proteolytic remodeling aligned with endogenous ovarian follicle development. In parallel, the non‐degradable annealing chemistry within and between microgels preserved overall scaffold integrity, producing a semi‐degradable structure that allowed tissue remodeling without premature scaffold collapse. Additionally, preparation of ovarian tissue as small cubes increased the tissue surface‐to‐volume ratio, increasing the tissue surface area presented to the surrounding MAP scaffold and the host environment. Thus, both tissue geometry and scaffold architecture likely contribute to the engineered host‐graft interface established by the OvaMAP transplantation strategy.

At 20 weeks, both non‐encapsulated grafts and OvaMAPs restored circulating estradiol, progesterone, and physiologic suppression of FSH, indicating integration of the grafts with the host HP axis. This timing aligns with clinical observations that ovarian function resumes 4 to 5 months after transplantation, supporting the translational relevance of our transplantation model. Estradiol levels in the non‐encapsulated group were highly variable, reflecting the well‐documented heterogeneity of human ovarian tissue and the unpredictable clinical duration of graft function, which ranges from months to years. In contrast, OvaMAP recipients exhibited more consistent estradiol levels, suggesting that controlled engineering of the host‐graft interface and the synthetic hydrogel environment may reduce tissue functional variability. Addressing this variability is a key unmet need in OTCT as outcome uncertainty complicates patient counseling and treatment planning.

Despite these promising results, several limitations should be taken into consideration. The non‐encapsulated and OvaMAP transplantation strategies differed in tissue presentation, with conventional tissue strips used for non‐encapsulated grafts and smaller tissue cubes incorporated within OvaMAPs, limiting our ability to distinguish the individual contributions of tissue geometry and MAP encapsulation to differences between these groups. The use of an immunodeficient xenograft model does not recapitulate the full spectrum of innate and adaptive immune responses that influence angiogenesis, fibrosis, and long‐term graft remodeling in patients. MAP hydrogels have been shown to modulate immune responses in other tissues [41, 42, 43, 44, 45, 46] and such interactions may contribute to graft performance but were not captured here. Furthermore, the identity and role of early infiltrating DAPI^+^/CD31^−^ cells remains undefined and warrants further investigation. While TER‐119^+^ RBCs were found within infiltrating host vessels, this immunolabeling represents a static indication of perfusion and does not provide dynamic information on the quality of blood flow. Real‐time imaging approaches capable of assessing graft perfusion in vivo would therefore strengthen future studies [20, 47, 48, 49]. Although this work focused on improving graft vascular integration, additional optimization of hydrogel chemistry may further enhance follicle survival, support later stages of folliculogenesis, or accelerate revascularization. Additionally, the MAP and bulk hydrogel formulations differed in mechanical properties, limiting our ability to distinguish the independent contributions of microporosity and scaffold mechanics to graft revascularization. Donor availability also limited sample size and age diversity; future studies across a broader donor population will be necessary to assess reproducibility.

Beyond their relevance to ovarian tissue, these findings highlight the host‐graft interface as an actionable therapeutic target for whole tissue transplantation. The modular architecture of MAP hydrogels provides a clinically adaptable platform that can be further tuned to incorporate proangiogenic cues [22, 50, 51] or retain endogenous angiogenic growth factors [27, 32]. As a fully synthetic and compositionally defined material, this system also offers advantages for manufacturing consistency and regulatory translation compared to naturally derived matrices.

In conclusion, engineering the host‐graft interface through the OvaMAP transplantation strategy enabled sustained, perfused revascularization of human ovarian tissue and supported long‐term endocrine function restoration within a clinically relevant timeframe. By stabilizing vascular remodeling and reducing functional variability, this approach addresses two central limitations of current OTCT practice: ischemic graft injury and unpredictable graft performance. Our results establish a foundation for biomaterial‐based strategies aimed at extending graft lifespan and improving the reliability of fertility and endocrine function restoration for patients at risk of POI.

## 4 Materials and Methods

### 4.1 Microgel Formulation

Similar to the chemistry described by Dumont et al. [52], poly(ethylene glycol) (PEG) microgels were generated using a precursor solution composed of 8‐arm PEG‐maleimide (PEG‐MAL) (40 kDa, > 90% purity, JenKem Technology, USA) crosslinked with a trifunctional plasmin‐sensitive crosslinking peptide (Ac‐GCYK↓NSGCYK↓NSCG, MW 1525.69 g/mol, > 90% Purity, GenScript, USA, ↓ indicates the cleavage site of the peptide) (YKNS). PEG‐MAL was pre‐functionalized with 1 mM of integrin‐binding peptide (Ac‐GCGYGRGDSGP, MW 1067.10 g/mol, > 85% Purity, GenScript, USA) (RGD). The final microgel precursor formulation consisted of 10 wt% PEG‐MAL, 1 mM RGD, and 2.77 mM YKNS, resulting in a 16:7 stoichiometric ratio of ‐MAL to ‐SH groups between PEG and YKNS. PEG and RGD were suspended in isotonic 50 mM HEPES buffer (pH = 7.4 at 20°C) and then mixed and allowed to react for 15 min at 20°C. Next, hydrochloric acid (HCl, 1N, Sigma‐Aldrich, USA) was thoroughly mixed into the PEG‐RGD solution to lower the solution's pH to 1.5. YKNS was then dissolved in 5 mM tris‐(2‐carboxyethyl)phosphine (TCEP) (Pierce Premium Grade TCEP‐HCl, Thermo Scientific, USA) and added to the mixture.

### 4.2 Microgel Generation

Microgels were generated using a step emulsification microfluidic device previously described by de Rutte et al. [53]. A master mold (SU‐8 silicon wafer with silanization) for step emulsification microfluidic devices with wedge geometry and channel height of 11 µm was fabricated by FlowJEM (FlowJEM Inc., Canada).To prepare microfluidic devices, polydimethylsiloxane (PDMS) base was mixed with crosslinker from the PDMS Sylgard 184 kit (Dow Silicones Corporation, USA) at a 10:1 mass ratio. The mixture was then degassed, poured over the master mold, and degassed again before being left to cure overnight at 80°C. Next, the cured PDMS was peeled from the SU‐8 silicon master mold, and microfluidic devices were cut from the PDMS replica cast. 1.5 mm holes were punched at the inlet and outlet ports of the PDMS microfluidic devices using a biopsy punch (Miltex Biopsy Punch, Electron Microscopy Sciences, USA). Devices and glass slides were then washed with isopropanol, followed by ethanol to remove dust. Finally, devices and glass slides were plasma etched for 1 min and bonded.

Prior to using devices for microgel generation, devices were treated with Rain‐X Glass Water Repellent (Illinois Tool Works, USA) for 1 min and rinsed with Novec 7500 oil (3M, USA). The treated devices were then placed in an 80°C oven for 1 h. The PEG‐MAL precursor solution was then injected into the microfluidic device at a flow rate of 1.2 mL/hr. The oil solution consisting of 1.5 wt% 008‐FluoroSurfactant (RAN Biotechnologies, USA) in Novec 7500 oil was injected into the device at a flow rate of 2.4 mL/hr. The generated microgels were collected in a conical tube and left at room temperature overnight to complete crosslinking.

The following day, crosslinked microgels were washed to remove the oil and surfactant. First, excess oil was aspirated from the bottom of the conical tube with a syringe. Next, a solution consisting of 20 wt% 1H,1H,2H,2H‐Perfluorooctanol (Thermo Scientific Chemicals, USA) in Novec 7500 oil was added to the remaining microgel solution. Then, isotonic 50 mM HEPES buffer (pH = 7.4 at 20°C) was added to the tube to swell and disperse the microgels, while hexanes were added to lower the Novec 7500 oil density. The tube was centrifuged at 2000 × g for 5 min prior to removing the supernatant and the hexanes wash was repeated three more times. Next, HEPES washes were performed five times. Microgels were washed with 70% ethanol for 10 min. Finally, microgels were washed two more times with HEPES buffer.

### 4.3 Microgel Size Distribution

Prior to washing, crosslinked microgels in Novec 7500 oil solution were imaged with brightfield on a transmitted light microscope. After washing, microgels in HEPES buffer were imaged with the same microscope settings once a day for 6 consecutive days. The diameters of 300–400 microgels for each group were manually measured in FIJI (ImageJ) analysis software [54].

### 4.4 MAP Hydrogel Fabrication

Microgels were formulated such that unreacted maleimide groups remained available after microgel crosslinking. Annealing of microgels into a MAP hydrogel was performed on the same day as microgel washing to prevent the remaining maleimide groups from undergoing hydrolytic degradation. Washed microgels were centrifuged at 18 000 × g for 5 min. The supernatant was removed, and pelleted microgels were then mixed at 10% v/v with a photoinitiator solution containing 600 mg/mL of Irgacure 2959 (2‐Hydroxy‐4′‐(2‐hydroxyethoxy)‐2‐methylpropiophenone, Sigma‐Aldrich, USA) in NVP (1‐Vinyl‐2‐pyrrolidinone, Sigma‐Aldrich, USA). Microgels were incubated for 5 min, followed by centrifugation at 18 000 × g for 5 min. The supernatant was removed, and pelleted microgels were pipetted into a cylindrical PDMS mold (4 mm diameter, 3.58 mm height) using a positive displacement pipette. Finally, microgels were exposed to ultraviolet light (λ = 365 nm, 1090 µW/cm^2^) for 3 min to form the MAP hydrogel through photoinitiated radical polymerization of remaining maleimide functional groups.

### 4.5 MAP Hydrogel Porosity

To characterize the porosity of the MAP hydrogel, microgels were fluorescently labeled by tagging the YKNS crosslinker with a fluorophore (CF488A Maleimide, Biotium) at a 1:600 stoichiometric ratio between ‐MAL in the dye and ‐SH in the crosslinker. Microgels and MAP hydrogels were then fabricated as described above. Multiple regions across 5 MAP hydrogels were subsequently imaged on a Leica SP8 laser scanning confocal microscope (85 µm z‐stack height, 5 µm step size). Porosity, pore size, and number of pores were quantified with FIJI (ImageJ) as previously described [55, 56]. Briefly, images were converted to 8‐bit format, thresholded using the Otsu algorithm, and smoothed with the built‐in smooth function (Figure S1A). Pores between the fluorescently labeled microgels were then analyzed with the Analyze Particles function. The analysis was limited to detect sizes ranging between 5 µm^2^ and Infinity. For each z‐slice, the analysis generated the total number of pores, the area of each pore, the total area occupied by pores, and the percent area occupied by pores. Only z‐slices containing in‐focus microgels and well‐defined thresholding were included in the analysis. Raw pore areas as well as theoretical pore diameters are reported. Theoretical pore diameters were derived by treating the raw pore areas as circles. The diameters of these circles with areas corresponding to the raw pore areas were then calculated.

### 4.6 Bulk Hydrogel Fabrication

Bulk hydrogels were fabricated using a precursor solution composed of 8‐arm PEG‐vinyl sulfone (PEG‐VS) (40 kDa, > 90% purity, JenKem Technology, USA) crosslinked with YKNS. PEG‐VS was pre‐functionalized with 0.75 mM RGD. The final bulk hydrogel formulation consisted of 7% PEG‐VS, 0.75 mM RGD, and 4.42 mM YKNS, resulting in a 1:1 stoichiometric ratio of ‐VS to ‐SH groups between PEG and YKNS. PEG and RGD were separately dissolved in isotonic 50 mM HEPES buffer (pH = 7.6 at 20°C) and then combined and left to react for 15 min at 20°C. YKNS was then dissolved in HEPES buffer and mixed with the PEG functionalized with RGD. The final hydrogel mixture was crosslinked for 5 min.

### 4.7 Hydrogel Mesh Size

The mesh size of MAP and bulk hydrogel formulations was theoretically estimated following a previously described method [57]. Briefly, two types of bulk hydrogels were prepared: (1) PEG‐MAL bulk hydrogel matching the MAP hydrogel formulation and (2) PEG‐VS bulk hydrogel. PEG‐MAL bulk hydrogels (50 µL) were left to crosslink overnight at 20°C in a humidity chamber, while PEG‐VS bulk hydrogels (50 µL) were left to crosslink for 5 min at 20°C. After crosslinking, hydrogels were submerged in Milli‐Q water and allowed to swell overnight at 37°C. The weight of the swollen hydrogels was then recorded. Next, hydrogels were lyophilized, and dry weights were recorded. Both swollen and dry hydrogel weights were subsequently used to calculate the mass swelling ratio with the following equation:

$$Q_{m}=\frac{swollen\;weight}{dry\;weight}$$

where *Q_m_* is the mass swelling ratio. Volumetric swelling ratio (*Q_v_*) was then calculated using the following equation:

$$Q_{v}=1+\left(\frac{\rho_{p}}{\rho_{s}}\times \left(Q_{m}-1\right)\right)$$

where ρ_*p*_ is the polymer density and ρ_*s*_ is the solvent density. Next, the average molecular weight between crosslinks (${\bar{M}}_{c}$) was calculated as described below:

$$\frac{1}{{\bar{M}}_{c}}=\frac{2}{{\bar{M}}_{n}}\;-\frac{\frac{\bar{\nu }}{V_{1}}\left(\ln\left(1-v_{2}\right)+v_{2}+\chi_{1}v_{2}^{2}\right)}{v_{2}^{1/3}-\frac{v_{2}}{2}}$$

where ${\bar{M}}_{n}$ is the number average molecular weight of polymer chains in the absence of crosslinker, *v* is the specific volume of the polymer, *V*_1_ is the molar volume of the solvent, *v*_2_ is the volume fraction of the polymer in the swollen state, which is equivalent to the reciprocal of *Q_v_*, and χ_1_ is the polymer‐solvent interaction parameter. The values for the defined parameters are shown in Table 1. ${\bar{M}}_{c}$ was then used to find the root mean square end‐to‐end distance of the polymer chain ($\sqrt{{\bar{r}}_{0}^{2}}$) which can be described by:

$$\sqrt{{\bar{r}}_{0}^{2}}=l\sqrt{C_{n}N}$$

where *l* is the average bond length between C‐C and C‐O bonds in the repeat unit of PEG [‐O‐CH_2_‐CH_2_‐], *C_n_* is the Flory characteristic ratio for PEG, and *N* is the number of links between monomers in the polymer chain. *N* can be further defined with ${\bar{M}}_{c}$ using the following equation:

$$N\;=2\frac{{\bar{M}}_{c}}{M_{r}}$$

where *M_r_* is the molecular mass of the PEG repeating unit. Finally, mesh size (ξ) was calculated as shown below.

$$\xi =Q_{v}^{1/3}\;\sqrt{{\bar{r}}_{0}^{2}}$$

**TABLE 1:** Parameters for Hydrogel Mesh Size Calculations.

Parameter	Description	Value
ρ_*p*_	Polymer density	1 g/cm^3^
ρ_*s*_	Solvent (water) density	1 g/cm^3^
${\bar{M}}_{n}$	Number average molecular weight of polymer chains in the absence of crosslinker	10 000 g/mol
$\bar{v}$	Specific volume of polymer	1 g/cm^3^
*V*_1_	Molar volume of solvent (water)	18 cm^3^/mol
χ_1_	Polymer‐solvent interaction parameter	0.426 [58]
*l*	Average bond length between C‐C and C‐O bonds in the repeat unit of PEG	1.46 nm [59]
*C_n_*	Flory characteristic ratio for PEG	4 [59]
*M_r_*	Molecular mass of PEG repeating unit	44 g/mol [59]

### 4.8 Mechanical Characterization

Rheological characterization of MAP and bulk hydrogels was performed using a TA Instruments HR30 Rheometer. Sandpaper was affixed to the bottom plate, and hydrogel samples (8 mm discs) were loaded onto the instrument. An amplitude sweep was conducted at a constant angular frequency of 10 rad/s over a strain range of 0.01–100% to determine the linear viscoelastic region (LVR). Based on the identified LVR, frequency sweeps were performed from 0.1 to 100 rad/s at a constant strain of 0.1% for MAP hydrogels and 1% for bulk hydrogels. For each hydrogel sample, the storage modulus was averaged across the frequency sweep to obtain a mean storage modulus, which was subsequently used for comparison between hydrogel formulations.

### 4.9 Hydrogel Diffusion

The diffusion of fluorescently labeled molecules through MAP and bulk hydrogels was assessed by analyzing the amount of molecule passing through the hydrogels. This was achieved using a framework composed of a cell culture insert and a 3D‐printed tube (Figure S1C). Preparation of hydrogel samples differed between the MAP and bulk hydrogel groups due to the malleable nature of MAP hydrogels. A MAP hydrogel sheet was prepared by annealing microgels in a square PDMS mold (10 × 10 mm in width and length, 2.7 mm in height). Next, a 3D‐printed square tube (7.35 × 7.35 mm in width and height, 150 mm in height) was used to cut out the prepared MAP hydrogel in the shape of the tube, and the MAP hydrogel‐tube construct was transferred to a cell culture insert (cellQART 12‐Well Cell Culture Insert, PET, 8.0 µm, Sterlitech, USA). In contrast, bulk hydrogel precursor solution was directly crosslinked inside the 3D‐printed tube prior to being transferred to a cell culture insert. 3D‐printed tubes were secured within cell culture inserts using a PDMS ring (12 mm outer diameter, 10 mm inner diameter) placed around each 3D‐printed tube. The cell culture inserts were then placed in a 12‐well plate containing 2 mL of DPBS per well and were left overnight at 37°C to allow hydrogels to swell and equilibrate.

The following day, the cell culture inserts were transferred to a fresh 12‐well plate containing 1.6 mL of DPBS per well. Cell culture inserts containing empty 3D‐printed tubes were used as controls. A solution containing a fluorescent molecule (2 µM) (Table 2) was then pipetted into the 3D‐printed tubes (*t*_0_). At specific time points, 100 µL of solution from the well beneath the cell culture insert was aspirated and collected in a 96‐well black plate (Non‐Treated Coverglass Surface, Thermo Scientific. USA) for analysis. Subsequently, the cell culture inserts were transferred to a fresh well within the 12‐well plate. The 12‐well plates were maintained at 37°C when solution samples were not being collected.

**TABLE 2:** Molecules Used for Hydrogel Diffusion Assay.

Molecule Type	Molecular Weight (kDa)	Conjugation	Product
Dextran	4	Fluorescein isothiocyanate	FD4, Sigma–Aldrich, USA
Dextran	40	Fluorescein isothiocyanate	FD40S, Sigma–Aldrich, USA
Bovine serum albumin	66	Alexa Fluor 488	A13100, Invitrogen, USA

Fluorescence intensities of all collected samples were measured after 6 and 96 h using a plate reader (Synergy H1 Multi‐Mode Microplate Reader, BioTek). The concentration of fluorescently labeled molecules in the collected samples was calculated using the measured fluorescence intensity and standard curves relating known concentrations to measured fluorescence intensities. Concentrations of fluorescent molecules in the collected samples were then converted to the amount of fluorescent molecules, which was subsequently used to calculate the cumulative diffusion of molecules over time. These values were then converted to percent molecule diffused over time using the amount of molecules contained in the original fluorescent molecule solution pipetted into the 3D‐printed tubes at *t*_0_. Finally, these percentage values for each fluorescent molecule were normalized to the terminal percent molecule diffused in the control condition.

### 4.10 Ethical Approval for the Procurement of Human Ovaries

As previously described [60, 61], whole human ovaries were obtained from deceased donors for non‐clinical research through the International Institute for the Advancement of Medicine (IIAM) and associated Organ Procurement Organizations (OPOs). IIAM and the associated OPOs comply with state Uniform Anatomical Gift Acts (UAGA) and are certified and regulated by the Centers for Medicare and Medicaid Services (CMS). These OPOs are members of the Organ Procurement and Transplantation Network (OPTN) and the United Network for Organ Sharing (UNOS) and operate under standards established by the Association of Organ Procurement Organizations (AOPO) and UNOS. Informed, written consent was obtained from the deceased donors’ families prior to tissue procurement for the tissue used in this study. A biomaterial transfer agreement is in place between IIAM and the University of Michigan that restricts the use of the tissue to pre‐clinical research not involving the fertilization of gametes. The use of deceased donor ovarian tissue in this research is categorized as “not regulated” per 45 CFR 46.102 and the “Common Rule” as it does not involve human subjects and therefore complies with the University of Michigan Institutional Review Board (IRB) requirements.

### 4.11 Human Ovarian Tissue Collection

Upon arrival in the laboratory, donor ovaries were transferred to pre‐cooled holding media composed of Quinn's Advantage Medium with HEPES (QAMH, ART‐1024, CooperSurgical, USA) supplemented with 10% Quinn's Advantage Serum Protein Substitute (SPS, ART‐3010, CooperSurgical, USA). The outer cortex layer was collected from the ovaries using a custom cutting guide (Reprolife, Japan), which produced cortical tissue squares measuring approximately 1 mm in thickness and 10 mm in both length and width. At this stage, a subset of cortex samples was fixed in Bouin's fixative (Ricca Chemical, USA). The remaining fresh cortical squares were cryopreserved using a previously published slow freezing protocol [38, 60, 61]. Briefly, tissue squares were placed into cryovials containing pre‐cooled cryoprotectant media composed of 10% SPS, 0.75 M dimethyl sulfoxide (DMSO, D2650, Sigma‐Aldrich, USA), 0.75 M ethylene glycol (102466, Sigma‐Aldrich, USA), and 0.1 M sucrose (S1888, Sigma–Aldrich, USA) in QAMH. Following equilibration at 4°C for 30 min, cryovials were slowly cooled from 4°C to −9°C at a rate of −2°C /min, manually seeded, held at −9°C for 4 min, and then slowly cooled to −40°C at a rate of −0.3°C/min. Finally, cryovials were submerged in liquid nitrogen and stored in a cryogenic storage dewar until further use.

### 4.12 Human Ovarian Tissue Characterization

The human ovarian tissue used in this study was obtained from two deceased donors (donors A and B). Both donors were of reproductive age (A: 29 years old; B: 20 years old) and did not have a medical history indicative of conditions that would affect ovarian function.

Donor follicle density was assessed throughout the ovarian cortex using the fresh tissue fixed in Bouin's fixative at the time of tissue collection. Samples were fixed at 4°C overnight and then washed in dH_2_O for 1 h, 50% ethanol for 1 h, 70% ethanol for 1 h, and finally stored in 70% EtOH at 4°C. Fixed samples were then embedded in paraffin at the University of Michigan Dental School Histology Core and serially sectioned at a thickness of 5 µm, producing four sections per slide for up to 50 slides per tissue sample. Every other slide was subsequently stained with hematoxylin and eosin (H&E) and imaged using a digital slide scanner (Leica Aperio AT2, Leica Biosystems, Germany) set to 20× magnification (Figure S3A) at the University of Michigan Unit for Laboratory Animal Medicine Pathology Core. Whole slide scans were imported into QuPath analysis software (v0.2.2) for manual follicle counting as described by Machlin et al. [62].

Briefly, every 8^th^ tissue section was analyzed for follicles, with a total of 24 sections analyzed for donor A and 10 sections analyzed for donor B. Analysis of every 8^th^ tissue section corresponded to a sampling spacing of 40 µm, which prevented duplicate counts of primordial, transitional primordial, or primary follicles. As such, every primordial, transitional primordial, and primary follicle was counted for each analyzed tissue section. Larger follicles were only counted where the nucleus was visible to prevent duplicate counts.

To calculate follicle density, tissue area was measured for all 24 and 10 sections for donors A and B, respectively, using FIJI (ImageJ). Tissue volume was then calculated by multiplying tissue section area by a tissue thickness of 20 µm rather than 40 µm to account for smaller primordial follicles that could have been omitted in between every 8^th^ tissue section that was analyzed. Follicle density for each analyzed tissue section was subsequently calculated as the total number of follicles in the tissue section divided by the tissue section volume. The calculated follicle densities for donors A and B were 48.05 ± 73.07 follicles/mm^3^ and 642.9 ± 95.28 follicles/mm^3^, respectively (Figure S3B).

### 4.13 Human Ovarian Tissue Thawing

Cortex squares were thawed following a previously described protocol [38, 60]. Briefly, cryovials containing cortex were removed from liquid nitrogen and left at room temperature for 30 s before being immersed in a 37°C water bath. Once the cryoprotectant medium had thawed (after approximately 1.5 min), the tissue was removed from the vial and placed into the first thawing solution (QAMH with 10% SPS, 0.5 M DMSO, 0.5 M ethylene glycol, 0.1 M sucrose) for 10 min. The tissue was then sequentially incubated in solutions with decreasing concentrations of cryoprotectants: the second thawing solution (QAMH with 10% SPS, 0.25 M DMSO, 0.25 M ethylene glycol, 0.1 M sucrose), the third thawing solution (QAMH with 10% SPS, 0.1 M sucrose), and the fourth thawing solution (QAMH with 10% SPS) for 10 min each. The fourth thawing solution served as a maintenance solution. All four solutions were equilibrated to room temperature for the thawing procedure.

### 4.14 Human Ovarian Tissue Preparation for Implantation

To prepare non‐encapsulated tissue for implantation, thawed cortex squares were manually cut into strips measuring approximately 1 mm in width and 5 mm in length. Similarly, to prepare tissue for encapsulation in bulk and MAP hydrogels, thawed cortex squares were manually cut into cubes measuring approximately 1 mm in length and width, thereby increasing the tissue surface‐to‐volume ratio by approximately 30%. All manual cutting was performed with tissues being maintained in the fourth thawing solution.

### 4.15 Human Ovarian Tissue Encapsulation in Hydrogels

MAP and bulk hydrogels were prepared with 4 tissue cubes per hydrogel. To encapsulate the tissue cubes in MAP hydrogels, 25 µL of the pelleted microgel/photoinitiator mixture was pipetted into a cylindrical PDMS mold (4 mm diameter, 3.58 mm height). The 4 tissue cubes were then placed on top of the pipetted microgels and covered with an additional 20 µL of microgels. Finally, the construct was exposed to ultraviolet light (λ = 365 nm, 1090 µW/cm^2^) for 3 min to produce human ovarian tissue cubes encapsulated in a MAP hydrogel (OvaMAP). To encapsulate the tissue in bulk hydrogels, 4 tissue cubes were placed in the cylindrical PDMS mold. Then, 45 µL of bulk hydrogel precursor solution was pipetted into the mold and allowed to crosslink for 5 min.

### 4.16 Ovariectomy and Intraperitoneal Implantation

All animal procedures in this work were performed in accordance with the protocol approved by the Institutional Animal Care and Use Committee (IACUC) at the University of Michigan (PRO00011284). First, a bilateral ovariectomy was performed on 10–11‐week‐old NOD.Cg‐*rkdc^scid^ Il2rg^tm1Wjl^*/SzJ (NSG) female mice (RRID:IMSR_JAX:005557, The Jackson Laboratory) to model primary ovarian insufficiency. Mice were anesthetized with isoflurane (2%–3%) via inhalation and were then administered preemptive analgesics (Carprofen, RIMADYL, Zoetis, USA, 5 mg/kg body weight) via subcutaneous injection. A longitudinal midline incision was then made in the abdominal wall using aseptic techniques and procedures. The intraperitoneal space was exposed, and the ovaries were removed by cauterizing the uterine horns directly below the ovary and cutting off the ovaries with microscissors. Next, the parametrial fat pads were exposed one at a time. One tissue strip or hydrogel (bulk or MAP with tissue cubes) was implanted in the fat pad by folding the fat around the tissue strip or hydrogel and sealing the fat pouch with absorbable 8‐0 sutures. As such, each mouse was implanted with either two tissue strips or two hydrogels (bulk or MAP). The fat pads containing tissue strips or hydrogels were then returned to the intraperitoneal cavity, and the incisions were closed in two layers (abdominal muscles and skin) using absorbable 5‐0 sutures and aseptic techniques and procedures. Mice were placed in a clean cage to recover and were given analgesics 24 h after the initial preemptive analgesic was administered. Mice were monitored postoperatively for 7–10 days and administered additional analgesics as needed.

### 4.17 Implant Removal

Implants were removed at the terminal time points of 3 days or 1, 3, 6, or 20 weeks. Mice were anesthetized as described above. A longitudinal midline incision was made in the abdominal wall, and the fat pads containing the tissue strips or hydrogels were excised and placed in 4% paraformaldehyde. Implants were left to fix overnight at 4°C before washing three times in DPBS for 1 h each and then storing in 70% ethanol at 4°C.

### 4.18 Human and Mouse Vasculature Visualization

We utilized tissue clearing techniques rather than conventional sectioning methods, which typically offer a limited view of samples and risk tearing hydrogel samples. Tissue clearing allowed us to analyze the implants and provided a comprehensive visualization of the interactions between the implants and the surrounding tissues of the host. However, due to variability in the amount of fat enveloping implants, implants were first sectioned to a consistent thickness of 800 µm using a vibratome (VF‐310‐0Z, Precisionary Instruments, USA). Next, a CUBIC tissue clearing protocol previously described by Matsumoto et al. [63] was adapted to clear the implant sections. The protocol consists of three distinct phases: (1) delipidation and decolorization, (2) staining, and (3) refractive index (RI) matching (Table 3). Briefly, delipidation and decolorization of the implant sections were initiated by immersing the sections in an equivalent volume mixture of dH_2_O and CUBIC‐L (10% (wt/wt) N‐butyldiethanolamine (B0725, TCI America, USA), 10% (wt/wt) Triton X‐100 (X100, Sigma‐Aldrich, USA), 80% (wt/wt) dH_2_O) with shaking at 37°C overnight. The next day, this mixture was replaced with fresh CUBIC‐L. CUBIC‐L was refreshed every 48 h for a total CUBIC‐L incubation duration of 10 days. At the end of 10 days, to stop the delipidation and decolorization process, sections were washed with 1X phosphate‐buffered saline (PBS) with shaking at room temperature for at least 2 h. This washing step was repeated more than three times.

**TABLE 3:** CUBIC Clearing and Staining Protocol Timeline.

Phase	Day	Step
Delipidation and Decolorization	1	1/2 CUBIC‐L
	2	CUBIC‐L
	4	Refresh CUBIC‐L
	6	Refresh CUBIC‐L
	8	Refresh CUBIC‐L
	10	Refresh CUBIC‐L
Staining	12	Wash + Block
	13	CD31
	16	Wash + UEA/Secondary/DAPI
RI matching	19	Wash + 1/2 CUBIC‐R+
	20	CUBIC‐R+
	21	Refresh CUBIC‐R+
	23	Image or store are room temperature

Next, of the sections that were cleared, one section from the center of each implant was selected for immunofluorescence staining. To stain the implants, sections were blocked with 5% normal goat serum (ab138478, Abcam, USA) in 1X PBS with shaking at 37°C overnight. Sections were then incubated in a solution consisting of a mouse‐specific vasculature primary antibody (CD31, 1:150, #77699, Cell Signaling Technology, USA) diluted in blocking solution with shaking at 37°C for 3 days. Following this step, sections were washed in blocking solution three times with shaking at room temperature for 1 h each. Next, sections were incubated in a solution containing a human‐specific vasculature marker, Ulex europaeus agglutinin 1 (UEA‐1, DyLight 649, 1:250, DL‐1068‐1, Vector Laboratories, USA), secondary antibody (Goat anti‐Rabbit, Alexa Fluor 555, 1:1000, A21428, Invitrogen, USA) and nuclear counterstain (DAPI, 1:1000, D1306, Invitrogen, USA) diluted in blocking solution with shaking at 37°C for 3 days. Finally, the samples were washed in 1X PBS three times with shaking at room temperature for 1 h each before proceeding to the RI matching phase.

To perform RI matching, sections were immersed in an equivalent volume mixture of dH_2_O and CUBIC‐R+(M) (45% (wt/wt) antipyrine (D1876, TCI America, USA), 30% (wt/wt) N‐methyl‐nicotinamide (M0374, TCI America, USA), 0.5% (vol/vol) N‐butyldiethanolamine, 24.5% (wt/wt) dH_2_O) with shaking at room temperature overnight. Next, this mixture was replaced with fresh CUBIC‐R+(M), and sections were incubated with shaking at room temperature overnight. The next day, CUBIC‐R+(M) was refreshed, and sections were incubated for an additional day. After this final CUBIC‐R+(M) incubation step, implant sections were ready for imaging.

Finally, sections were imaged on a Leica SP8 laser scanning confocal microscope. Tilescans composed of Z‐stacks of 500 µm with a z‐step of 2.5 µm were acquired for each implant section. Laser power and gain were maintained across all implant sections.

### 4.19 Human and Mouse Vasculature Quantification

The percent area positive for human UEA‐1 and mouse CD31 within human ovarian tissue was measured in 36 implants (12 non‐encapsulated tissue strips, 12 OvaMAPs, 12 bulk hydrogels). For each implant, one 800 µm‐thick vibratome section was imaged to produce a 500 µm‐tall confocal image containing 200 z‐slices. Ultimately, 20 z‐slices were analyzed for each implant using FIJI (ImageJ) in order to quantify human and mouse vasculature within human ovarian tissue.

To obtain the percent area measurements, the unaltered confocal image for each of the 36 implants was first converted from Leica's LIF file format into BigTIFF file format using the FIJI Bio‐Formats plugin. The BigTIFF image was then trimmed from a 200 z‐slice image to a 100 z‐slice image by generating a substack containing every other z‐slice. This 100 z‐slice substack was then divided into five substacks containing 20 z‐slices each. From these five substacks, one representative substack was selected for analysis. For the selected substack, regions of interest (ROIs) were then outlined on each z‐slice around either the non‐encapsulated tissue strip or the hydrogel‐encapsulated tissue cubes.

The ensuing steps were performed for the human UEA‐1 and mouse CD31 channels separately. To subtract background signal, the mean gray value was measured in one 50 × 50 µm high background signal ROI for each z‐slice within the substack. The mean value of these 20 measurements was then subtracted from each z‐slice within the substack. Next, all 20 z‐slices were smoothed with the built‐in smooth function and thresholded using the Moments algorithm. The percent area positive for either human UEA‐1 or mouse CD31 was then obtained for each z‐slice using the Analyze Particle function. The analysis was constrained to the previously outlined ROIs. Additionally, the analysis was limited to detect sizes ranging between 19.635 µm^2^ and Infinity. The lower limit of 19.635 µm^2^ was applied assuming a minimum vessel diameter of 5 µm. For each z‐slice, the Analyze Particles function output the percent area positive for human UEA‐1 or mouse CD31. Finally, for each implant, the reported percent area positive for human UEA‐1 or mouse CD31 corresponded to the mean value of the 20 analyzed z‐slices.

The characterization of mouse CD31 vasculature through quantification of vessel length density and mean branch length was performed using the Quantitative Vascular Analysis Tool (Q‐VAT) macro [64] for FIJI. Only non‐encapsulated tissue strip and OvaMAP confocal images were subjected to this quantification since the previous percent area measurements revealed no infiltration of mouse CD31 vasculature within the bulk hydrogel implants. To perform the analysis, Q‐VAT requires two‐dimensional images. As such, the same substacks used for the previous percent area measurements were utilized to generate maximum intensity projections. However, because of graft geometry variations along the 20‐slice depth, only the first 10 or last 10 z‐slices were used to generate maximum intensity projections, ensuring that accurate ROIs delineating only human ovarian tissue could be generated. No background subtraction was performed on these z‐slices. The maximum intensity projections were then processed using the Q‐VAT masking and analysis tools using the parameter inputs listed in Table 4. ROIs were outlined around either the non‐encapsulated tissue strip or the encapsulated tissue cubes to restrict the Q‐VAT masking and analysis to the grafts rather than the surrounding mouse tissue.

**TABLE 4:** Q‐VAT Input Parameters.

Parameter	Input
Calibration (µm/pixel)	1.136
Radius of biggest object (µm)	40
Particle size lower range (µm^2^)	10000
Radius for median filtering (µm)	2
Remove small particles (µm^2^)	10
Thresholding method	Otsu
Vascular compartment separation threshold (µm)	10
Close labels radius (µm)	10
Prune ends threshold (µm)	5

### 4.20 Functional Mouse Vasculature Visualization

Visualization of functional mouse vasculature infiltrating OvaMAPs was achieved by staining for a mouse‐specific red blood cell marker. OvaMAPs were sectioned at a thickness of 400 µm using a vibratome. Next, sections were incubated in a permeabilization solution composed of 0.2% Triton X‐100 (Sigma‐Aldrich, USA) in 1X PBS with shaking at 37°C overnight. The following day, sections were blocked with 5% normal goat serum in 1X PBS with shaking at 37°C overnight. Then, sections were incubated in a solution containing a mouse‐specific red blood cell primary antibody (TER‐119, 1:200, 14‐5921‐85, Invitrogen, USA) diluted in blocking solution with shaking at 37°C for 3 days. Following this step, sections were washed in blocking solution three times with shaking at room temperature for 1 h each. Next, sections were incubated in a solution containing secondary antibody (Goat anti‐Rat, Alexa Fluor Plus 647, 1:1000, A48265, Invitrogen, USA) and nuclear counterstain (DAPI, 1:1000, D1306, Invitrogen, USA) diluted in blocking solution with shaking at 37°C for 3 days. Sections were then washed in 1X PBS three times with shaking at room temperature for 1 h each. In order to mount the sections for imaging, the sections were then subjected to the RI matching steps used in the CUBIC clearing protocol. Finally, sections were imaged on a Leica SP8 laser scanning confocal microscope.

### 4.21 Serum Hormone Analysis

At the terminal time points of 3 days and 1, 3, 6, and 20 weeks, blood was collected via cardiac puncture. Following blood collection, blood samples were stored at 4°C overnight and then centrifuged at 2000 × g for 15 min. Blood serum was collected and stored at −80°C until analysis. Serum samples were analyzed at the Ligand Assay and Analysis Core of the Center for Research in Reproduction at the University of Virginia. Follicle‐stimulating hormone was analyzed using an in‐house ELISA protocol with a lower detection limit of 0.016 ng/mL. Estradiol was analyzed using an ELISA (Estradiol – Mouse and Rat Serum, Plasma & Tissue Homogenates, American Laboratory Products Co., USA) with a lower detection limit of 5 pg/mL. Progesterone was analyzed using an ELISA (Progesterone – Mouse and Rat Serum, Plasma & Tissue Homogenates, Immuno‐Biological Laboratories, USA) with a lower detection limit of 0.15 ng/mL.

### 4.22 Artificial Intelligence Use

During the preparation of this manuscript, the authors used the University of Michigan GPT‐4.1 to proofread sentences and paragraphs for spelling, grammar, and clarity. The platform was used for language refinement, not for content generation. All suggestions were reviewed and edited by the authors prior to incorporation into the manuscript.

### 4.23 Statistical Analysis

All statistical analysis was performed in GraphPad Prism 10. Unless otherwise noted, data is expressed as mean ± standard deviation (mean ± SD). Error bars throughout figures are mean ± standard deviation per group. Data in Figures 1 and 3 were analyzed by one‐way ANOVA with Tukey's multiple comparisons test, *p* < 0.05, different letters indicate statistical significance. Data in Figures 2 and 4 were analyzed by two‐way ANOVA with Šidák's multiple comparisons test, p < 0.05, significant differences are indicated as ^*^ *p* ≤ 0.05, ^**^ *p* ≤ 0.01, ^***^ *p* ≤ 0.001, ^****^ *p* ≤ 0.0001. Data in Figure 6 were analyzed by the Kruskal–Wallis test with Dunn's multiple comparisons test, *p* < 0.05, significant differences are indicated as ^*^ *p* ≤ 0.05, ^**^ *p* ≤ 0.01, ^***^ *p* ≤ 0.001.

## Author Contributions

Conceptualization and Ideation: DIP, VP, BMB, AS; Study design: DIP, VP, BMB, AS; Material Characterization DIP, CEF, MAJ, DSS, EET; Murine Experiments: DIP, MAR, CEF, DSS, BV, MAB; Image Acquisition and Analysis DIP, CEF, MAR; Manuscript Drafting: DIP, AS; Manuscript Review & Editing: DIP, SCLP, VP, BMB, AS.

## Funding

Eunice Kennedy Shriver National Institute of Child Health and Human Development grants R01HD104173 (AS), R01HD122991 (AS, BMB), T32HD079342 (DSS, MAB), Z1A HD009005 (MAB); National Institute of Biomedical Imaging and Bioengineering grant R01EB030474 (BMB); National Institute of Dental and Craniofacial Research grant T32DE007057 (DIP, MAR, BV, MAB); National Science Foundation Graduate Research Fellowship Program DGE1256260 (MAR), DGE1841052 (EET); University of Michigan Rackham Predoctoral Fellowship Program (EET).

## Conflicts of Interest

The authors declare no conflicts of interest.

## Supporting information




**Supporting File**: advs78523‐sup‐0001‐SuppMat.docx.

## Data Availability

The data that support the findings of this study are available from the corresponding author upon reasonable request.
