# Supplementary material for: Engineering the Host‐Graft Interface Stabilizes Vascular Integration and Improves Endocrine Function of Transplanted Ovarian Tissue
Source: Adv Sci (Weinh). 2026 Nov 11:e78523. Online ahead of print. doi: 10.1002/advs.78523 (PMC13755366; doi:10.1002/advs.78523)
Supplement: Supplementary file 1 [file ADVS-9999-e78523-s001.docx]

Supporting Information

Engineering the host-graft interface stabilizes vascular integration and improves endocrine function of transplanted ovarian tissue

*Despina I. Pavlidis^1^, Monica A. Rionda^1^, Chloe E. Fischer^1^, Maria A. Jennings^1^, Delaney S. Sinko^1^, Bhavya Vats^1^, Emily E. Thomas^1^, Margaret A. Brunette^1,2^, Sasha Cai Lesher-Pérez^1,3,4^, Vasantha Padmanabhan^5,6,7^, Brendon M. Baker^1^, Ariella Shikanov* ^1,5,8,9^*

**Figure S1. Characterization of MAP Hydrogel Porosity, Mesh Size, and Diffusion. (A)** Workflow illustrating porosity quantification from confocal images using FIJI (ImageJ). **(B)** Nanoporous bulk hydrogel mesh sizes for MAP hydrogel and bulk hydrogel formulations (n = 6 per condition) were estimated from swelling ratios. **(C)** Illustration of experimental methods used to determine the diffusion of molecules through bulk and MAP hydrogels. Short- and long-term diffusion of 4 kDa FITC-DEX (**D)**, 40 kDa FITC-DEX **(E)**, and 66 kDa Alexa Fluor 488-BSA **(F)** through MAP (pink) and bulk (purple hydrogels) (n = 3 per hydrogel type). Short-term measurements were conducted hourly for 6 hours while long-term measurements were conducted every 24 hours over 96 hours.

**Figure S2. Hydrogel Storage Moduli.** Storage modulus of PEG-VS bulk hydrogel, PEG-MAL MAP hydrogel, and bulk hydrogel fabricated using the PEG-MAL microgel formulation. A minimum of n = 6 hydrogels were tested per condition, error bars are mean ± standard deviation per group. Data analyzed by one-way ANOVA with Tukey’s multiple comparisons test, p < 0.05, significant differences indicated as ****p ≤ 0.0001.

**Figure S3. Human Donor Ovarian Cortex Characterization. (A)** Representative hematoxylin and eosin (H&E) staining of fresh fixed ovarian cortex from donors A and B, with corresponding estimated follicle densities **(B)**. Error bars are mean ± standard deviation per group.

**Figure S4. Human Vasculature Regression within Bulk Hydrogel-Encapsulated Tissue.** Percent area of implanted, bulk hydrogel-encapsulated human ovarian tissue cubes that stained positively for human vasculature. Data represent individual grafts from distinct mouse hosts (n = 3 per time point), error bars are mean ± standard deviation per group. Data analyzed by one-way ANOVA with Tukey’s multiple comparisons test, p < 0.05, different letters indicate statistical significance.
